# Serum and blister‐fluid elevation and decreased epidermal content of high‐mobility group box 1 protein in drug‐induced Stevens–Johnson syndrome/toxic epidermal necrolysis†

**DOI:** 10.1111/bjd.17610

**Published:** 2019-03-26

**Authors:** D.F. Carr, C.‐W. Wang, T. Bellón, L. Ressel, G. Nwikue, V. Shrivastava, W. Bergfeld, A.L. Jorgensen, W.‐H. Chung, M. Pirmohamed

**Affiliations:** ^1^ Department of Molecular and Clinical Pharmacology University of Liverpool Liverpool U.K.; ^2^ Department of Dermatology Drug Hypersensitivity Clinical and Research Center Chang Gung Memorial Hospital Keelung, Linkou, Taipei Taiwan; ^3^ College of Medicine Chang Gung University Taoyuan Taiwan; ^4^ La Paz University Hospital Health Research Institute (IdiPAZ) Madrid Spain; ^5^ Departments of Veterinary Pathology and Public Health University of Liverpool Liverpool U.K.; ^6^ Naval Medical Center Portsmouth Portsmouth VA U.S.A.; ^7^ Department of Dermatology and Dermatopathology Cleveland Clinic Foundation Cleveland OH U.S.A.; ^8^ Departments of Biostatistics University of Liverpool Liverpool U.K.

## Abstract

**Background:**

High‐mobility group box 1 (HMGB1) is a damage‐associated molecular‐pattern protein. Stevens–Johnson syndrome (SJS)/toxic epidermal necrolysis (TEN) are serious, immune‐mediated skin‐blistering conditions.

**Objectives:**

To determine serum and/or blister‐fluid total HMGB1 levels in SJS/TEN cohorts, and HMGB1 expression in formalin‐fixed, paraffin‐embedded (FFPE) SJS/TEN skin vs. healthy and maculopapular exanthema (MPE) skin.

*Methods* Serum HMGB1 was quantified in Malawian nevirapine‐induced hypersensitivity, Taiwanese SJS/TEN and Spanish SJS/TEN cohorts. FFPE skin (healthy skin, MPE, SJS/TEN) was stained and assessed for HMGB1 expression.

**Results:**

Serum total HMGB1 was not significantly elevated in patients with nevirapine‐induced SJS/TEN (3·98 ± 2·17 ng mL ^−1^), MPE (3·92 ± 2·75 ng mL ^−1^) or drug reaction with eosinophilia and systemic symptoms (4·73 ± 3·00 ng mL ^−1^) vs. tolerant controls (2·97 ± 3·00 ng mL ^−1^). HMGB1 was significantly elevated in Taiwanese patients with SJS/TEN, highest during the acute phase (32·6 ± 26·6 ng mL ^−1^) vs. the maximal (19·7 ± 23·2 ng mL ^−1^; *P* = 0·007) and recovery (24·6 ± 25·3 ng mL ^−1^; *P* = 0·027) phases. In blister fluid from Spanish patients with SJS/TEN, HMGB1 (486·8 ± 687·9 ng mL ^−1^) was significantly higher than in serum (8·8 ± 7·6 ng mL ^−1^; *P* <0·001). Preblistered SJS/TEN skin showed decreased epidermal nuclear HMGB1 expression in upper epidermis vs. healthy or MPE skin but retained basal/suprabasal expression.

**Conclusions:**

Epidermal HMGB1 expression was decreased in SJS/TEN skin. Retained basal/suprabasal epidermal HMGB1 expression may exacerbate localized injury in SJS/TEN.

## Abstract 2

**What's already known about this topic?**

- High‐mobility group box 1 (HMGB1) is a marker of tissue injury and innate immune response.
- Elevation of serum HMGB1 in patients with serious cutaneous adverse drug reactions has been reported previously.
- To date, no analysis of HMGBI distribution in Stevens–Johnson syndrome/toxic epidermal necrolysis (SJS/TEN) patient blister‐fluid and skin biopsy tissue has been described.

**What does this study add?**

- This study confirms serum HMGB1 elevation in SJS/TEN in three independent cohorts.
- It reports, for the first time, super‐elevation of HMGB1 in blister fluid and significantly decreased epidermal expression in preblistered SJS/TEN skin with suprabasal retention.
- These results are highly distinct from healthy or maculopapular exanthema skin and may represent a viable diagnostic tool.

**Linked Comment:** Dodiuk‐Gad. *Br J Dermatol* 2019; **181**:18–19.

Plain language summary available online

Respond to this article

Stevens–Johnson syndrome (SJS)/toxic epidermal necrolysis (TEN) is characterized by blistering of the skin and mucosal membranes with significant areas of skin detachment (up to 10% for SJS and > 30% for TEN). Although rare (6·52 cases per million patient years),^1^ SJS/TEN can have a severe impact on patients, with a significant mortality rate and many suffering from long‐term debilitating sequelae, including blindness. In addition, SJS/TEN can be a resource burden on healthcare systems, with the average hospital stay estimated at 7–12·6 days, of which 1·7–4·9 days are spent in intensive treatment units.^2^

Drugs are among the most common causes of SJS/TEN. A number of predictive genetic markers for drug‐specific SJS/TEN have been identified.^3^ Several serum protein markers are elevated in patients with SJS/TEN at the time of the reaction and have the potential to act as prognostic or diagnostic markers. These include granulysin, Fas ligand and, more recently, interleukin (IL)‐15.^4^, ^5^, ^6^ However, Nakajima *et al*. suggested that while granulysin and Fas ligand are possible candidate biomarkers,^7^ the duration of elevation is limited and therefore false‐negative results for SJS/TEN are a possibility.

High mobility group box 1 (HMGB1) is an example of a damage‐associated molecular pattern molecule, which is critical in linking cell death to inflammation and in the progression of disease. HMGB1 sits at the intersection between infectious and sterile inflammation. It is actively released in an acetylated form from activated immune cells and passively released in the nonacetylated form during necrotic cell death.^8^ Previous studies have shown that total HMGB1 is significantly elevated in serum from patients with drug reaction with eosinophilia and systemic symptoms (DRESS)^9^ and drug‐induced SJS,^7^ at the time of the reaction and may discriminate between serious cutaneous drug eruptions and milder phenotypes [maculopapular exanthema (MPE)].

The aim of this study was to assess total HMGB1 levels in serum samples from three independent cohorts of patients with SJS/TEN, as well as from blister fluid in a subset of patients. We also evaluated HMGB1 expression levels and distribution in formalin‐fixed, paraffin‐embedded (FFPE) skin biopsy samples from patients with SJS/TEN.

## Patients and methods

Samples from three independent SJS/TEN patient cohorts were brought together for the purpose of this study.

### Nevirapine patient cohort

Patients were prospectively recruited, as previously described, after informed consent was obtained.^10^ The study was approved by the research ethics committees of the College of Medicine, Malawi, and Liverpool School of Tropical Medicine, Liverpool, U.K. Briefly, the study recruited 1117 antiretroviral‐naive HIV‐positive patients from the Queen Elizabeth Central Hospital, Blantyre, Malawi, between March 2007 and December 2008. All were self‐reported black African, over the age of 16, years and had no baseline jaundice. All patients were diagnosed as having clinical stage 3/4 HIV or had a CD4^+^ count < 250 cells μL^−1^ and were treated with fixed‐dose nevirapine (NVP), lamivudine and stavudine with follow‐up over 26 weeks. CD4^+^ counts and liver function tests (LFTs) were monitored at 0, 6, 14 and 18 weeks. A total of 51 patients presented with NVP‐induced cutaneous hypersensitivity fulfilling the criteria of one or more of the following phenotypes: (i) MPE with no systemic manifestations and improvement only on stopping treatment; (ii) DRESS – widespread rash with systemic manifestations (i.e. fever, cough or abnormal LFTs); (iii) SJS – extensive rash affecting two or more mucous membranes or blistering eruptions affecting < 10% of body surface area (BSA); (iv) TEN – blistering rash affecting > 30% of BSA and two or more mucous membranes. Patients with 10–30% affected BSA had the overlap syndrome.

Each of the 51 patients with hypersensitivity were age‐ and sex‐matched to two NVP‐tolerant HIV‐positive controls (*n* = 102). Blood samples were taken from patients prior to commencement of NVP treatment (0 weeks) and then at 2, 6, 10, 14, 18 and 20 weeks. In patients in whom a hypersensitivity reaction occurred, a sample was taken at the time of the first presentation (acute reaction) and no further sample taken thereafter as the patient was discontinued from the study. For comparison to adverse reaction samples, tolerant control samples at the 2‐week time point were used. Serum was isolated and frozen at –80 °C. The purpose of analysing this cohort was to determine variability in serum HMGB1 levels between different types of cutaneous adverse drug reactions during the acute phase vs. tolerant controls.

### Taiwanese Stevens–Johnson syndrome/toxic epidermal necrolysis cohort

A total of 73 Han Chinese patients with SJS/TEN were enrolled from 2009 to 2015 at Chang Gung Memorial Hospital (CGMH) in Taiwan (Table S1; see Supporting Information); they had received referrals from other national hospitals (National Taiwan University Hospital, Taichung Veterans General Hospital, National Cheng Kung University Hospital, Kaohsiung Medical University, Chung‐Ho Memorial Hospital). SJS/TEN was characterized by rapid development of blistering exanthema with purpuric macules, accompanied by mucosal involvement and skin detachment. Diagnosis of SJS/TEN was assessed according to phenotypes based on the criteria of the RegiSCAR study group.^11^, ^12^, ^13^ The study was approved by the institutional review board (IRB) and ethics committee of CGMH, based on Taiwanese law (IRB no. 97‐0509B, no. 100‐4657A3, no. 103‐2562C, no. 105‐3600C). Serum samples were obtained from patients during the acute, maximum and recovery stages of SJS/TEN and stored at –80 °C. The time to onset of illness in each patient was also evaluated. The acute stage was defined as < 6 days from onset of illness; the maximum stage was defined as the time from onset to maximal skin detachment (without progression of skin detachment); and the recovery stage was defined as the time to complete skin healing. The purpose of analysing this cohort was to determine serum HMGB1 not only in the acute phase of SJS/TEN, but also at the maximal point of the reaction and during the recovery phase (both of which were determined retrospectively).

### Spanish Stevens–Johnson syndrome/toxic epidermal necrolysis and drug reaction with eosinophilia and systemic symptoms cohort

Patients (*n* = 23) diagnosed with SJS, TEN, SJS/TEN or DRESS/SJS/TEN overlap were identified in different hospitals in Madrid, Spain, belonging to the PIELenRed consortium. SJS/TEN was characterized by purpuric macules and/or target‐like lesions, accompanied by mucosal involvement and skin detachment. Patients were classified according to the consensus criteria (Table S2; see Supporting Information).^14^ DRESS was diagnosed according to the RegiSCAR scoring system.^15^ Patient serum and blister‐fluid samples were obtained at first presentation (acute) and stored at –80 °C. The study was approved by the Research Ethics Committee of Príncipe de Asturias University Hospital (the coordinating centre of the PIELenRed Consortium). The purpose of analysing this cohort was to compare HMGB1 levels during the acute phase of SJS/TEN in both serum and blister fluid.

### Formalin‐fixed, paraffin‐embedded skin biopsy tissue samples

FFPE skin samples (healthy controls, *n* = 5; drug‐induced rash, *n* = 7; SJS/TEN, *n* = 7) were identified from the histology archive database at the Cleveland Clinic, OH, U.S.A., from 2013 to 2016 (accessed January 2017). An internal diagnosis or description search included the terms ‘Stevens‐Johnson syndrome’ or ‘toxic epidermal necrolysis’ and drug eruption/drug reaction (including ‘dermal hypersensitivity reaction’). Specific cases were selected where a diagnosis of drug‐induced SJS/TEN or MPE was very strongly favoured and was supported by the clinical notes. For normal healthy control samples, normal skin from excision specimens was used. Suspected causal drugs were identified from clinical notes.

### Serum high mobility group box 1 measurement

All serum samples were transferred to the Wolfson Centre for Personalised Medicine, University of Liverpool, U.K., for analysis. Total HMGB1 protein concentrations in serum and blister fluid (where available) were determined by enzyme‐linked immunosorbent assay according to the manufacturer's protocol (Oxford Biosystems, Oxford, U.K.). Spectrophotometric analysis at 450 nm was undertaken using a DTX880 Plate Reader (Beckman Coulter, High Wycombe, U.K.). Blister fluid samples were analysed iteratively, firstly neat and then at 30‐fold and 300‐fold dilution in phosphate buffered saline until quantifiable within the detection range of the assay. Sample measurements failing to reach the manufacturer's lower limit of quantification (0·2 ng mL^−1^) or where replicates were discordant by > 15% were excluded.

### High mobility group box 1 immunohistochemistry

For immunohistochemistry (IHC), sections were dewaxed and subjected to antigen retrieval in Dako PT Buffer High pH (Agilent Technologies, Abingdon, U.K.) using a computer‐controlled antigen‐retrieval workstation (PT Link; Agilent Technologies) for 20 min at 98 °C. Sections were then stained in an automated immunostainer (Link 48; Agilent Technologies), using a primary antibody against human HMGB1 [rabbit polyclonal, ab18256, 1 in 1000 (Abcam, Cambridge, U.K.)] and incubated for 1 h at room temperature (RT). This was followed by a 30‐min incubation at RT with the polymer peroxidase‐based detection system (Anti Mouse/Rabbit Envision Flex+; Agilent Technologies). The reaction was visualized with diaminobenzidine (Agilent Technologies). Consecutive sections incubated with nonimmune rabbit serum served as negative controls. The positive reaction was represented by a distinct brown nuclear (or rarely cytoplasmic) reaction. Positive control was represented by epidermis and follicle in normal skin.

Semi‐quantitative analysis of immunohistochemical HMGB1 expression intensity was done by a pathologist with respect to distribution within the epidermal layers and also to nuclear staining, as follows: negative stain (–) was defined as the absence of a specific brown stain; minimal stain (+/–) was judged as a barely visible specific brown stain; moderate stain (+) was judged as clear evident staining that was milder than the intensity of the keratinocytes of normal skin; strong stain (++) was of an intensity comparable with keratinocytes of the normal skin (positive control). Stain intensity was determined according to the most represented pattern in the biopsy available on the slide.

### Statistical analysis

Statistical analysis was carried out with Prism 5 (GraphPad, La Jolla, CA, U.S.A.) and RStudio version 1·1·414. To analyse the difference in HMGB1 levels between serum and blister fluid in the Spanish cohort and difference in HMGB1 levels with time in the Taiwanese cohort, linear mixed models were fitted using the *lmer* function in R package *lme4*. This approach allowed for more than one measurement from the same individual. For the Spanish cohort, HMGB1 levels were log‐transformed to ensure model residuals were normally distributed. Comparison of data between unpaired sample groups (i.e. between hypersensitivity phenotypes) was undertaken using a Kruskal–Wallis test. A threshold cut‐off, based on the recently reported 97·5% quantile reference ranges,^16^ was used to classify samples as elevated (> 2·3 ng mL^−1^). Analysis of the resulting binary outcome between phenotype groups was by the χ^2^‐test. To adjust for multiple testing, the Bonferroni approach was undertaken, adjusting the *P*‐value threshold for nominal significance (0·05) by the number of tests. As not all outcomes tested were independent (e.g. combined cutaneous hypersensitivity phenotype and the specific hypersensitivity reaction phenotype groups), we believe this to be a conservative approach.

## Results

### Serum and blister fluid high mobility group box 1 concentrations

#### Nevirapine‐induced cases and controls

**Figure 1:**
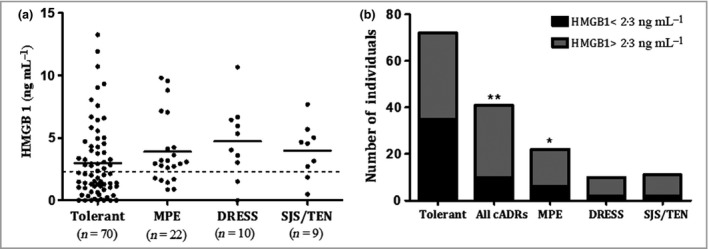
Serum total high mobility group box 1 (HMGB1) concentration in the Malawian, nevirapine‐exposed HIV cohort at time of reaction. (a) Concentration for different cutaneous hypersensitivity phenotypes (2 weeks after drug initiation for tolerant). The dashed line indicates the defined upper limit of normal (ULN; 2·3 ng mL ^−1^). (b) Number of individuals whose serum concentration was above (black bars) or below the ULN (grey bars). Statistical significance, determined by (a) Kruskal–Wallis test and (b) χ^2^, is indicated by *P < 0·05 and **P < 0·01. cADRs, cutaneous adverse drug reactions; MPE, maculopapular exanthema; DRESS, drug reaction with eosinophilia and systemic symptoms; SJS/TEN, Stevens–Johnson syndrome/toxic epidermal necrolysis.

Of the initially identified 51 patients with NVP cutaneous hypersensitivity and 102 tolerant controls, sera from 40 cases (22 MPE, nine DRESS, nine SJS/TEN) and 70 controls at time of reaction were available for analysis of total HMGB1 (Fig. 1). Using the Kruskal–Wallis test, we found that mean ± SD concentrations of total HMGB1 in each of the three NVP‐induced cutaneous phenotypes (MPE 3·92 ± 2·75 ng mL^−1^, DRESS 5·25 ± 2·65 ng mL^−1^, SJS/TEN 3·98 ± 2·17 ng mL^−1^) and combined (4·17 ± 2·81 ng mL^−1^) were higher (but not significantly) than those in the tolerant control group (2·97 ± 3·00 ng mL^−1^; *P* > 0·05) (Fig. 1a).

Using the χ^2^‐test, we found that the percentage of individuals with elevated levels above our defined upper limit of normal (ULN; > 2·3 ng mL^−1^) was higher in the combined cutaneous hypersensitivity (75%, *P* = 0·002), MPE (72%, *P* = 0·027), DRESS (89%, *P* = 0·029) and SJS/TEN (78%, *P* = 0·042) groups than in the tolerant group (46%). Only the combined cutaneous hypersensitivity phenotype remained significant after correction for multiple testing (*P* < 0·012).

#### Taiwanese patients with Stevens–Johnson syndrome/toxic epidermal necrolysis

**Figure 2:**
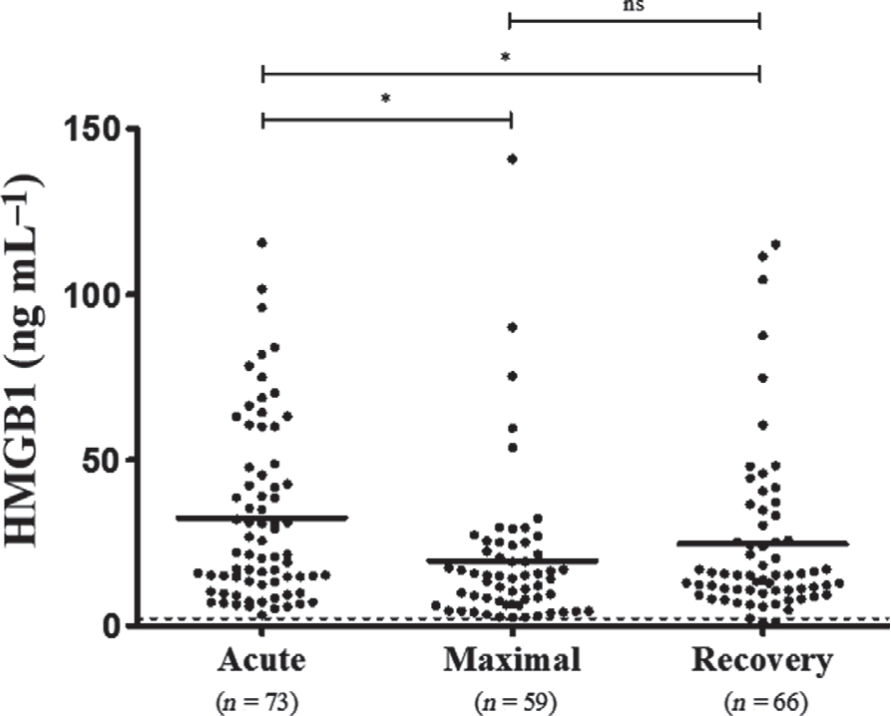
Total high mobility group box 1 (HMGB1) in serum from the Taiwanese SJS/TEN cohort during the acute phase of the reaction; maximal point of reaction and during the recovery phase. The dashed line indicates the notional upper limit of normal (2·3 ng mL ^−1^). Statistical significance determined by linear mixed modelling is indicated by ***P < 0·001. NS, nonsignificant.

Serum from 73 Taiwanese patients with SJS/TEN was analysed for HMGB1 (Fig. 2). This included 73 acute reaction samples, 59 samples at time of maximal reaction and 66 samples from the recovery phase. The mean ± SD total serum HMGB1 during the acute phase of the reaction was 32·6 ± 26·6 ng mL^−1^, which was higher than at both the maximal reaction time (19·7 ± 23·2 ng mL^−1^; *P* < 0·001) and recovery (24·6 ± 25·3 ng mL^−1^; *P* < 0·001). Both these differences were statistically significant after applying Bonferroni adjustment (*P*‐value threshold 0·017 for three pairwise comparisons). No statistically significant difference was observed between maximal reaction and recovery.

#### Spanish patients with Stevens–Johnson syndrome/toxic epidermal necrolysis

**Figure 3:**
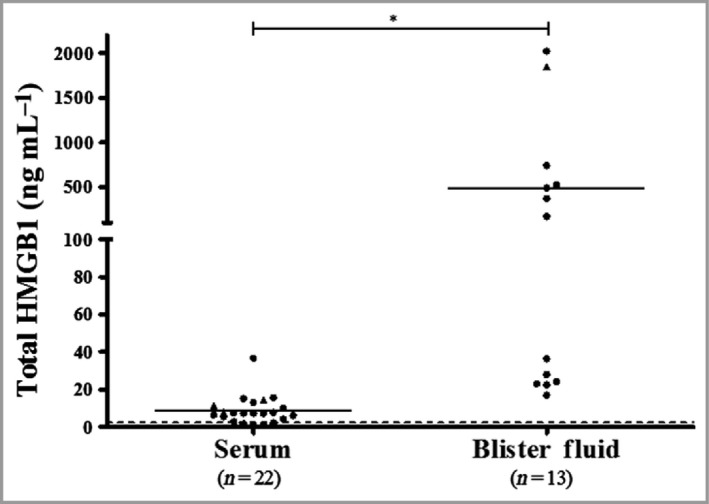
Total high mobility group box 1 (HMGB1) in serum and blister fluid from the Spanish SJS/TEN cohort at time of reaction. The dashed line indicates the notional upper limit of normal (2·3 ng mL ^−1^). Statistical significance determined by linear mixed modelling is indicated by ***P < 0·001. Circles represent SJS/TEN cases and triangles drug reaction with eosinophilia and systemic symptoms/TEN overlap.

Mean ± SD serum total HMGB1 (*n* = 22) in the Spanish SJS/TEN cohort was 8·8 ± 7·6 ng mL^−1^ (Fig. 3). Blister fluid levels (*n* = 13) were significantly higher at 486·8 ± 687·9 ng mL^−1^ (*P* < 0·001) than serum levels. In the 12 individuals from whom samples from both sites were available, there was no correlation between matched serum and blister‐fluid HMGB1 levels (*R* ^*2*^ = 0·036; data not shown).

### Skin tissue expression of high mobility group box 1

**Figure 4:**
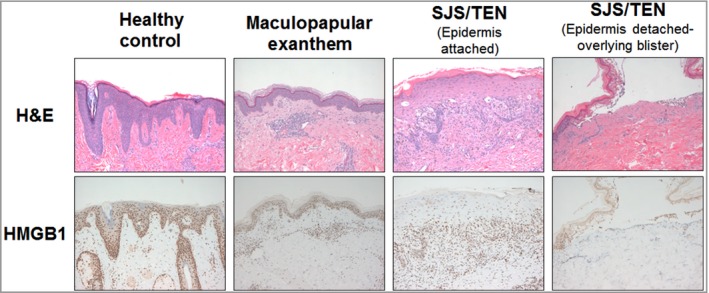
Immunohistochemical staining (×100) of high mobility group box 1 (HMGB1) in healthy, maculopapular exanthema and Stevens–Johnson syndrome/toxic epidermal necrolysis (SJS/TEN) skin. H&E, haematoxylin and eosin.

**Figure 5:**
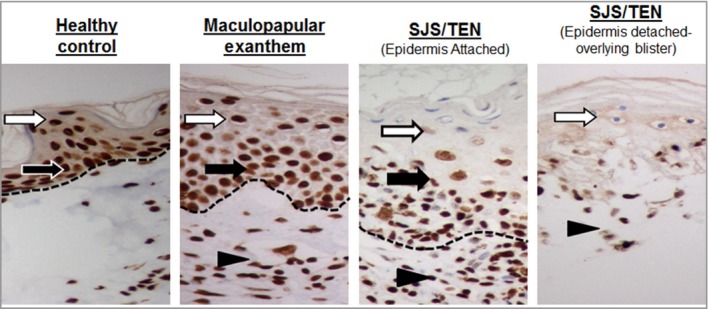
High mobility group box 1 staining (×400) at the epidermal basal/suprabasal layer in healthy, maculopapular exanthema and Stevens–Johnson syndrome/toxic epidermal necrolysis skin. Black arrow indicates the stratum basale; white arrow the stratum spinosum and arrowhead the inflammatory infiltrate. The dashed line indicates the dermal/epidermal junction.

Immunohistochemical staining of FFPE skin sections showed that, compared with healthy control and MPE skin, patients with SJS/TEN exhibited significantly less nuclear expression of total HMGB1 in the epidermis of nonblistered SJS/TEN skin (Fig. 4). Indeed, expression appeared to be limited to the basal/suprabasal layer (Fig. 5), an observation that was consistent in all individuals with SJS/TEN, regardless of the site of biopsy (Table 1). Prominent HMGB1 expression was also observed in infiltrating inflammatory cells in the skin from patients with MPE and with SJS/TEN.

**Table 1:** Semi‐quantitative analysis of high mobility group box 1 (HMGB1) immunohistochemistry (IHC) staining in healthy, maculopapular exanthema (MPE) and Stevens–Johnson syndrome/toxic epidermal necrolysis (SJS/TEN) skin biopsy sections from the Cleveland Clinic archival cohort

Phenotype	Biopsy site	Age (years)	Sex	Causal drug	IHC HMGB1 Expression				
					Epidermis	Nuclear stain within epidermis	Follicle/adnexae	Dermis	Inflammatory infiltrate
Healthy	Thigh	23	F	NA	++	All layers	++	+/–	NP
Healthy	Forehead	19	M	NA	++	All layers	++	+	NP
Healthy	Arm	61	F	NA	++	All layers	++	+/–	NP
Healthy	Back	58	F	NA	++	All layers	++	+/–	NP
Healthy	Inferior breast	40	F	NA	++	All layers	NP	+	NP
MPE	Arm	81	F	Vancomycin	+	All layers	++	+	++
MPE	Arm	61	M	Meropenem	++	All layers	++	+/–	++
MPE	Arm	59	F	Simvastatin	++	All layers	++	+/–	++
MPE	Arm	22	M	Aripiprazole	++	All layers	++	+/–	++
MPE	Scalp	78	F	Undetermined	++	All layers	++	+/–	++
MPE	Abdomen	59	F	Levetiracetam	++	All layers	++	+	++
MPE	Abdomen	62	M	Piperacillin/tazobactam	++	All layers	++	+	++
SJS	Back	62	M	Ceftaroline	+/–	Basal/suprabasal layer	++	+/–	++
SJS	Arm	51	M	Vancomycin	+/–	Basal/suprabasal layer	NP	+/–	++
SJS	Lip	23	F	Sulfamethoxazole/trimethoprim	+/–	Basal/suprabasal layer	NP	+	++
SJS/TEN	Thigh	54	M	Vancomycin	+/–	Basal/suprabasal layer	++	+/–	++
SJS/TEN	Abdomen	39	F	Piperacillin/tazobactam	+/–	Basal/suprabasal layer	++	+/–	++
TEN	Arm	24	F	Ketorolac	+/–	Basal/suprabasal layer	+/–	–	++
TEN	Chest	28	F	Sulfamethoxazole/trimethoprim	+/–	Basal/suprabasal layer	++	+/–	++

Intensity: (–) denotes negative; (+/–) denotes minimal; (+) denotes moderate; (++) denotes strong. F, female; NA, not applicable; NP, not present in the biopsy section analysed; M, male.

## Discussion

The data from this study show that total serum HMGB1 levels are elevated in patients with SJS/TEN, with levels being higher in blister fluid than in paired serum samples. Furthermore, the data highlight the changes occurring in lesional skin from patients with SJS/TEN, with HMGB1 expression being seen in the basal/suprabasal layer but reduced overall in the epidermis. However, the elevation in serum HMGB1 may be confounded by underlying disease as the effect in Malawian patients with advanced HIV disease was absent when compared with non‐HIV‐positive patients.

In our NVP hypersensitivity cohort from Malawi, although HMGB1 levels at the time of reaction in patients with SJS/TEN and DRESS were elevated, they were not significantly higher than those in both tolerant patients and patients with MPE (Fig. 1a). Creation of a binary outcome with a threshold cut‐off (> 2·3 ng mL^−1^) (Fig. 1b) provided some indication that HMGB1 may be able to discriminate between phenotypes to a small degree, but this is unlikely to be useful clinically. By contrast, in HIV‐negative patients with SJS/TEN from both Taiwan and Spain, serum HMGB1 levels were significantly elevated, with levels in the Spanish cohort (8·8 ng mL^−1^) similar to that previously observed in Japanese patients with SJS/TEN at the time of the reaction.^7^, ^9^ The HMGB1 concentrations were much higher in Taiwanese patients (36·2 ng mL^−1^; Fig. 2) than in the Spanish patients; the reasons for this unclear, but given the small sample sizes, it is perhaps best not to overinterpret the differences as these may be due to sample collection and storage differences rather than true biological variability. One variable that can be discounted is inconsistency in the SJS/TEN phenotype definition as both the Spanish and Taiwanese cohorts used the same criteria (RegiSCAR), while in the NVP cohort from Malawi the diagnostic criteria were closely aligned. It is important to note that the three independent cohorts studied were brought together retrospectively, from studies of differing design, to address different questions relating to HMGB1 elevation. This limits our ability to assess differences between the different cohorts.

The different results obtained from HIV‐positive patients from Malawi vs. those from HIV‐negative patients from Taiwan and Spain may be related to the confounding effect of HIV infection. Our Malawian patients were at an advanced stage of HIV infection (HIV clinical stage 3/4 and/or had a CD4^+^ count < 250 cells μL^−1^). HIV infection has previously been shown to cause elevated HMGB1 serum levels,^17^ while HMGB1 itself may enhance HIV replication.^18^ By contrast, HMGB1 levels tend to be reduced by antiretroviral therapy (ART), albeit long‐term therapy;^19^ few data are available in the short term, particularly from those at more advanced stages of HIV disease.^20^ Given the confounding effects of HIV disease and concomitant ART, it is perhaps not surprising that the total HMGB1 levels were lower than those seen in the Taiwanese and Spanish patients, and did not sufficiently distinguish between controls, patients with MPE and patients with SJS/TEN. Indeed, a significant 46% of tolerant controls exhibited HMGB1 serum levels above our nominal ULN (> 2·3 ng mL^−1^), which further highlights the potential influence of co‐medication and comorbidity on serum HMGB1 levels. It is also important to acknowledge that elevation in serum HMGB1 is not specific to cutaneous hypersensitivity reactions, and has been observed in HIV, epilepsy and a number of other inflammatory and autoimmune diseases.^17^, ^21^, ^22^

Serum HMGB1 levels in Taiwanese patients with SJS/TEN were significantly higher in the acute phase of the reaction than in both the maximal and recovery phases (Fig. 2), suggesting activation of this pathway at the time of presentation, indicative of tissue damage before treatment (symptomatic and specific) is given, may lead to alterations in levels. Interestingly, for the first time, we have shown that blister‐fluid levels of HMGB1 in patients with SJS/TEN were 55‐fold higher than in serum, indicating that voiding of HMGB1 from the keratinocytes in the epidermis is likely to contribute to the significantly elevated blister fluid levels (Fig. 3).

Our data from skin biopsies showed previously unreported decreased expression of nuclear HMGB1 in the epidermis of preblistered SJS/TEN skin coupled with the expression of HMGB1 in the basal and suprabasal layers (Fig. 5). Although this observation has been made in a small sample (*n* = 7) of biopsies from patients with SJS/TEN, the consistency of the finding across all patients would suggest that it is a true observation (Table 1). Keratinocyte death is likely to explain the decrease in epidermal expression, and subsequent increased levels of circulating serum total HMGB1. However, given that, histologically, SJS/TEN is characterized by separation of the epidermis and dermis, the role of HMGB1 retained at the suprabasal layer warrants further investigation. The dramatic difference between epidermal distribution of HMGB1 in SJS/TEN vs. control and MPE skin makes IHC analysis of HMGB1 a potential diagnostic marker for SJS/TEN, but this would require further investigation. Interestingly, our data also suggest that keratinocyte release of HMGB1 in SJS/TEN skin may occur prior to epidermal detachment (Fig. 4), and thus HMGB1 serum elevation may actually precede it, raising the possibility that it may represent a good early marker of cell death/tissue damage in SJS/TEN. Again, this will need further study.

Our study is limited by the fact that we have measured total HMGB1 but not its post‐translationally modified HMGB1 acetylated isoform. In its hyperacetylated form, HMGB1 can be actively secreted from multiple cell types (e.g. macrophages, natural killer cells, dendritic cells) as part of an innate immune response.^23^ Additionally, in its unacetylated form, it can be passively secreted from necrotic or damaged cells, with both mechanisms leading to significantly elevated extracellular HMGB1 levels.^24^ Given the activation of the innate immune response in SJS/TEN,^25^ activated macrophages are likely to be a source of HMGB1 (in its acetylated form).^26^ It is entirely plausible that elevation of unacetylated serum HMGB1 (passive released from dying keratinocytes) may differentiate between patients with SJS/TEN and individuals with milder phenotypes (MPE, DRESS) where widespread cell/tissue injury is not observed. This will require careful assessment of the HMGB1 isoforms, which we plan to undertake in the near future through the development of a new assay.

Our data suggest that, unlike granulysin and IL‐15,^6^ total HMGB1 serum levels do not correlate with SJS/TEN severity scores (SCORTEN; data not shown). This coupled with the observation that levels remain high even in the recovery phase suggests that its utility as a prognostic marker is likely to be limited (Fig. 2). Furthermore, confounding by other concomitant conditions such as HIV infection may lead to changes in HMGB1 levels, which may be difficult to interpret in relation to the occurrence of SJS/TEN. However, it is important to note that HMGB1 is associated with different physiological functions related to the innate immune response and dependent on its redox state.^26^, ^27^ Consequently, HMGB1 may be important in the pathogenesis of the keratinocyte damage seen in SJS/TEN, exacerbated by the infiltration of activated innate and adaptive immune cells, and the subsequent localized release of cytotoxic proteins, including granulysin, to augment keratinocyte injury. This is perhaps suggested by our skin biopsy analyses, but will require further work to understand fully the role of HMGB1 in the pathogenesis of SJS/TEN.

In conclusion, this study has shown that HMGB1 is elevated in patients with SJS/TEN, with levels being higher in blister fluid than in serum, but this may not be the case in all patient groups, particularly where the underlying concomitant disease (e.g. advanced HIV disease, as in our Malawian patients) and its treatment can affect HMGB1 levels. Our data from skin biopsies also, for the first time, demonstrate that HMGB1 expression is depleted in the epidermis of patients with SJS/TEN, with some HMGB1 being retained in the basal layer, in proximity to the epidermal/dermal junction, providing suggestive evidence of a putative role for HMGB1 in the pathogenesis of epidermal separation, although further work will need to be undertaken to elucidate this.

## Supporting information


**Table S1** Demographic and Clinical Data for the Taiwanese SJS/TEN Cohort.
**Table S2** Demographic and Clinical Data for the Spanish SJS/TEN Cohort.
