# Supplementary material for: Serum and blister‐fluid elevation and decreased epidermal content of high‐mobility group box 1 protein in drug‐induced Stevens–Johnson syndrome/toxic epidermal necrolysis
Source: Br J Dermatol. 2019 Mar 26;181(1):166–74. doi: 10.1111/bjd.17610 (PMC6617791; doi:10.1111/bjd.17610)
Supplement: Supplementary file 1 — Table S1 Demographic and Clinical Data for the Taiwanese SJS/TEN Cohort. Table S2 Demographic and Clinical Data for the Spanish SJS/TEN Cohort. [file BJD-181-166-s001.docx]

**SUPPLEMENTARY DATA**

**Supplementary Table 1.** Demographic and Clinical Data for the Taiwanese SJS/TEN Cohort. * denotes deceased individuals.

| **Patient No** | **Phenotype** | **SCORTEN** | **Age** | **Gender** | **Serum Sample Analysed** | | | **Causal_Drug** |
| --- | --- | --- | --- | --- | --- | --- | --- | --- |
|  |  |  |  |  | **Acute** | **Maximal** | **Recovery** |  |
| 1 | TEN | 2 | 50 | F | Y | Y | Y | sulindac, mephenoxalone |
| 2 | SJS | 0 | 25 | F | Y | Y | Y | oxcarbazepine |
| 3 | TEN | 2 | 41 | F | Y | Y | Y | carbamazepine |
| 4 | SJS | 2 | 46 | M | Y | Y | Y | unknown |
| 5 | SJS | 1 | 72 | M | Y | Y | Y | carbamazepine |
| 6 | TEN | 1 | 6 | F | Y | Y | Y | ibuprofen, ketoprofen |
| 7 | TEN | 3 | 87 | M | Y | Y | Y | unknown |
| 8 | SJS | 1 | 25 | F | Y | Y | Y | carbamazepine |
| 9 | SJS | 1 | 40 | F | Y | Y | Y | ibuprofen, amoxicillin |
| 10 | TEN | 2 | 48 | F | Y | Y | Y | unspecified NSAID |
| 11 | SJS | 2 | 43 | M | Y | Y | Y | carbamazepine |
| 12 | SJS | 1 | 45 | F | Y | N | Y | carbamazepine |
| 13 | TEN | 3 | 29 | F | Y | Y | Y | norfloxacin, ibuprofen, diclofenac |
| 14 | SJS | 1 | 35 | F | Y | Y | Y | carbamazepine |
| 15 | SJS | 2 | 51 | F | Y | Y | Y | nystatin, co-amoxiclav |
| 16 | SJS | 3 | 58 | M | Y | Y | Y | carbamazepine |
| 17 | SJS/TEN* | 4 | 76 | M | Y | Y | N | allopurinol |
| 18 | SJS | 1 | 61 | M | Y | Y | Y | phenytoin |
| 19 | SJS | 3 | 74 | M | Y | Y | Y | Unknown |
| 20 | SJS | 1 | 58 | F | Y | Y | Y | carbamazepine |
| 21 | SJS | 1 | 53 | F | Y | Y | Y | unknown |
| 22 | SJS | 1 | 31 | F | Y | Y | Y | metronidazole, unknown drug |
| 23 | SJS/TEN* | 1 | 50 | M | Y | Y | Y | allopurinol |
| 24 | SJS | 1 | 80 | F | Y | Y | Y | sulfamethoxazole |
| 25 | SJS/TEN | 0 | 31 | M | Y | Y | Y | omeprazole |
| 26 | SJS/TEN* | 2 | 49 | F | Y | Y | N | mecobalamine |
| 27 | SJS | 2 | 42 | M | Y | Y | Y | phenytoin |
| 28 | SJS/TEN | 2 | 49 | F | Y | Y | Y | carbamazepine |
| 29 | SJS | 1 | 63 | F | Y | N | Y | carbamazepine |
| 30 | TEN | 3 | 45 | F | Y | Y | Y | allopurinol |
| 31 | SJS | 1 | 75 | F | Y | Y | Y | etoricoxib |
| 32 | SJS | 0 | 38 | M | Y | Y | Y | chlormeaznone/paracetamol |
| 33 | SJS | 2 | 64 | M | Y | N | Y | allopurinol |
| 34 | SJS | 0 | 31 | M | Y | N | Y | allopurinol |
| 35 | TEN* | 4 | 41 | F | Y | Y | N | allopurinol |
| 36 | SJS | 1 | 66 | F | Y | Y | Y | allopurinol |
| 37 | SJS | 0 | 9 | M | Y | N | Y | carbamazepine |
| 38 | SJS | 3 | 72 | M | Y | N | Y | allopurinol |
| 39 | SJS | 1 | 51 | M | Y | N | Y | carbamazepine |
| 40 | SJS | 1 | 58 | F | Y | Y | Y | phenytoin |
| 41 | SJS | 1 | 40 | F | Y | Y | Y | naproxen, ofloxacin |
| 42 | SJS | 3 | 86 | M | Y | N | Y | allopurinol |
| 43 | SJS | 1 | 70 | F | Y | N | Y | phenytoin |
| 44 | SJS* | 3 | 82 | F | Y | Y | N | allopurinol, tramadol, chlorzoxazone, pentoxifylline, zolpidem, paracetamol, piroxicam, cephanmycin, tipepidine, ambroxol |
| 45 | SJS | 1 | 53 | M | Y | N | Y | carbamazepine |
| 46 | SJS | 2 | 70 | F | Y | N | Y | allopurinol |
| 47 | SJS/TEN | 1 | 47 | F | Y | Y | Y | carbamazepine |
| 48 | SJS | 4 | 83 | M | Y | Y | Y | allopurinol |
| 49 | SJS | 2 | 48 | M | Y | N | Y | phenytoin |
| 50 | SJS | 0 | 36 | M | Y | N | Y | allopurinol |
| 51 | SJS | 1 | 55 | M | Y | Y | Y | esomeprazole |
| 52 | SJS | 0 | 40 | F | Y | Y | Y | lamotrigine |
| 53 | TEN | 2 | 41 | M | Y | Y | Y | nevirapine |
| 54 | SJS | 2 | 53 | F | Y | Y | Y | ibuprofen aceclofenac |
| 55 | SJS | 1 | 77 | F | Y | Y | Y | ketorolac, sulindac |
| 56 | SJS | 1 | 72 | M | Y | Y | Y | carbamazepine |
| 57 | SJS | 1 | 34 | M | Y | Y | Y | esomeprazole |
| 58 | SJS | 1 | 75 | F | Y | Y | Y | ibuprofen, paracetamol/chlorzoxazone/caffeine/thiamine disulfide |
| 59 | SJS | 1 | 56 | F | Y | Y | Y | ticagrelor |
| 60 | SJS | 3 | 47 | M | Y | Y | Y | sulindac |
| 61 | SJS | 1 | 53 | M | Y | N | Y | sulfamethoxazole |
| 62 | SJS | 1 | 46 | F | Y | Y | Y | sulfamethoxazole |
| 63 | SJS | 1 | 86 | F | Y | Y | Y | allopurinol |
| 64 | SJS | 2 | 84 | F | Y | Y | Y | phenytoin |
| 65 | SJS/TEN* | 4 | 82 | F | Y | Y | N | levofloxacin, ceftazidime, fluconazole |
| 66 | TEN* | 4 | 53 | M | Y | Y | N | unspecified NSAID |
| 67 | SJS | 1 | 61 | F | Y | Y | Y | esomeprazole, geniflozacin |
| 68 | SJS/TEN* | 4 | 80 | F | Y | Y | N | esomeprazole, cephalexin, ceclofenac, vancomycin, ceftazidime |
| 69 | SJS | 1 | 13 | F | Y | Y | Y | ibuprofen, amoxicillin, co-amoxiclav |
| 70 | SJS | 0 | 34 | M | Y | Y | Y | fluconazole |
| 71 | TEN | 5 | 56 | M | Y | Y | Y | unknown |
| 72 | SJS | 1 | 46 | F | Y | Y | Y | carbamazepine |
| 73 | SJS | 3 | 65 | M | Y | Y | Y | meloxicam, mosapride, lercanidipine, telmisartan |

**Supplementary Table 2.** Demographic and Clinical Data for the Spanish SJS/TEN Cohort. * denotes deceased individuals.

| **Patient No** | **Phenotype** | **Age** | **Gender** | **Acute Serum** | **Blister Fluid** | **Causal Drug** |
| --- | --- | --- | --- | --- | --- | --- |
| 1 | SJS | 2 | M | N | Y | co-amoxiclav, ibuprofen |
| 2 | SJS | 34 | F | Y | N | lamotrigine |
| 3 | SJS* | 57 | M | Y | Y | ethambutamol, rifampicin, levetiracetam, amoxicillin |
| 4 | SJS | 54 | F | Y | N | amoxicillin, dexketoprofen |
| 5 | SJS* | 50 | M | Y | Y | sulfamethoxazole |
| 6 | TEN | 68 | M | Y | Y | vemurafenib |
| 7 | TEN | 41 | F | Y | Y | allopurinol |
| 8 | SJS | 89 | M | Y | N | azitromycin, N-acetylcysteine paracetamol/codeine, furosemide, levofloxacin, omeprazole |
| 9 | TEN | 43 | M | Y | Y | olsetamivir |
| 10 | SJS/TEN | 77 | M | Y | N | allopurinol |
| 11 | SJS/TEN | 50 | F | Y | Y | None |
| 12 | SJS | 35 | M | Y | Y | oxypurinol |
| 13 | SJS/TEN | 45 | F | Y | N | mirtazapine, vancomycin, ciprofloxacin, alprazolam, metoclopramide, ceftriaxone |
| 14 | Overlap DRESS, SJS/TEN | 43 | F | Y | Y | benznidazol |
| 15 | TEN | 48 | F | Y | Y | sulfamethoxazole |
| 16 | SJS/TEN | 41 | F | Y | N | sulfasalazine |
| 17 | SJS | 93 | M | Y | N | sulfamethoxazole |
| 18 | TEN | 5 | F | Y | Y | penicillin, amoxicillin |
| 19 | SJS/TEN* | 85 | F | Y | Y | cefuroxime, levofloxacin |
| 20 | SJS/TEN | 51 | F | Y | Y | None |
| 21 | SJS/TEN | 76 | F | Y | N | nabumetone, dexketoprofen, ciprofloxacin, cefazolin |
| 22 | Overlap DRESS, SJS/TEN | 25 | M | Y | N | benznidazol |
| 23 | Overlap DRESS, SJS/TEN | 16 | F | Y | N | lamotrigine |
